# Introducing the Rapid Alert Supply Network Extractor (RASNEX) tool to mine supply chain information from food and feed contamination notifications in Europe

**DOI:** 10.1371/journal.pone.0254301

**Published:** 2021-07-27

**Authors:** Marc C. Lorenzen, Armin A. Weiser, Robert Pieper, Monika Lahrssen-Wiederholt, Jorge Numata

**Affiliations:** German Federal Institute for Risk Assessment, Berlin, Germany; University of New South Wales (UNSW Sydney), AUSTRALIA

## Abstract

**Background:**

During food or feed contamination events, it is of utmost importance to ensure their rapid resolution to minimize impact on human health, animal health and finances. The existing Rapid Alert System for Food and Feed (RASFF) is used by the European Commission, national competent authorities of member countries and the European Food Safety Authority to report information on any direct or indirect human health risk arising from food or feed, or serious risks to animal health or the environment in relation to feed. Nevertheless, no methods exist to to collectively evaluate this vast source of supply chain information.

**Methods:**

To aid in the extraction, evaluation and visualization of the data in RASFF notifications, we present the Rapid Alert Supply Network Extractor (RASNEX) open-source tool available from https://doi.org/10.5281/zenodo.4322555 freely. Among RASNEX’s functions is the graphical mapping of food and feed supply chain operators implicated in contamination events. RASNEX can be used during ongoing events as a support tool for risk analysis using RASFF notifications as input.

**Results:**

In a first use case, we showcase the functionality of RASNEX with the RASFF notification on a 2017/2018 contamination event in eggs caused by the illegal use of fipronil. The information in this RASFF notification is used to visualize nine different flows of main and related food products. In a second use case, we combine RASFF notifications from different types of food safety hazards (*Salmonella spp*., fipronil and others) to obtain wider coverage of the visualized egg supply network compared to the first use case. Actors in the egg supply chain were identified mainly for Italy, Poland and Benelux. Other countries (although involved in the egg supply chain) were underrepresented.

**Conclusions:**

We hypothesize that biases may be caused by inconsistent RASFF reporting behaviors by its members. These inconsistencies may be counteracted by implementing standardized decision-making tools to harmonize decisions whether to launch a RASFF notification, in turn resulting in a more uniform future coverage across European food and feed supply chains with RASNEX.

## Introduction

Globalization of the agri-food industry has become very extensive. Flows of food and feed products as parts of supply chains involve a considerable number and diversity of entities, processes and locations. Fast-moving consumer goods such as chicken (*Gallus gallus domesticus*) eggs and chicken egg products are particularly challenging with respect to food and feed safety. Many chemical and biological agents can compromise egg and egg product safety, coupled to notorious difficulties in their back- and forward-tracing along flows of food and feed. The lifespan of fresh chicken eggs in the market is typically between 14 and 21 days, depending on the type of egg and the retailer. This requires fast reaction times to prevent direct or indirect risks to human health in case of entry of undesirable substances, contaminants, physical or biological agents into the flow of products. In 2002, European legislation laid down the general principles and requirements of the food law and established a system for reporting health and nutritional risks of foods among member countries: the Rapid Alert System for Food and Feed (RASFF) (Reg (EC) 178/2002) [1]. Whenever a RASFF member has information on a direct or indirect human health risk arising from food or feed, or serious risks to animal health or the environment in relation to feed [2, 3], they have an obligation to inform others using RASFF. All European Union (EU) Member States, European Economic Area (EEA) countries, the European Free Trade Association (EFTA) secretariat (coordinating the input from the EEA countries), Switzerland, the European Food Safety Authority (EFSA) and the European Commission (as a manager of the system) are members of RASFF and are automatically recipients of any RASFF alert. In this system, national contact points have a central position, verifying and completing RASFF notifications where necessary and forwarding them to the European Commission (DG SANTE). No comparable all-encompassing alert system exists at a worldwide level. A RASFF notification includes product descriptions, samplings and laboratory analyses, involved operators of the flow of food or feed products and taken measures for products and related products (among other information). As an example, a total of 4,118 original notifications and 10,388 follow-up notifications (containing additional information on measures taken and outcomes of investigations transmitted by control authorities) were transmitted through RASFF in 2019 [4]. One of the significant challenges of the RASFF system is the amount of data produced. Although the information shared can be valuable in crises, it has to be somehow aggregated, processed, visualized and interpreted before decisions can be made during ongoing contamination events. Here, the question arises of how to extract relevant data from RASFF notifications despite manual extraction, how to visualize affected supply chains and how to aggregate data in order to support decision making. To our knowledge, no general tool for this purpose exists.

Here we present a general-purpose software tool for the national food and feed control authorities: The Rapid Alert Supply Network Extractor (RASNEX). RASNEX 1.0 can extract all actors involved in an ongoing or past chemical contamination event or biological agent outbreak from the European RASFF notifications and compile related information in sheets, thereby enabling the use of this data for further analysis with other tools (e.g. FoodChain-Lab [5], RACE [6] or other future tools). RASNEX generates a graphical mapping of all actors of the supply network known and extracted by the software and involved in contamination events. RASNEX is programmed in the KNIME framework [7] and makes use of FoodChain-Lab developed by the German Federal Institute for Risk Assessment (BfR) [5]. RASNEX can be used to 1) extract and visualize food and feed supply chain information from a single RASFF notification related to a single ongoing (or past) contamination event and 2) visualize flows of food and feed products reported to be involved in food or feed contamination events during the past years by combining information from several contamination events. This information may be used as a “contrast medium” to identify otherwise invisible parts of trade networks and reveal inconsistent reporting behaviors of the member countries. We address the question whether it is possible to extract data from a single RASFF notification in order to map an ongoing contamination event. Our second research question is whether it is possible to automatically map at least parts of food or feed supply chains by using data from the RASFF system. Furthermore, we explore whether there are different reporting behaviors amongst member countries and what are the current limitations of the RASFF system.

## Background

Previously, others have employed statistical techniques to analyze RASFF notifications [8]. Analyzing the RASFF notifications related to food contact materials (FCMs), De Leo et al. [9] showed a significant association between the origin of the notified products and other categorical variables (hazard category, type of FCMs, notification type, distribution status and risk decision). Jan Mei Soon [10] analyzed RASFF for food fraud coming from China and found artificial enhancement to be the most common food fraud category affecting cereals, fruits & vegetables and dietetic foods & supplements. Others have used network analysis at the nation state level, e.g. Petroczi et al. [11] who focused on summarizing mycotoxin transgressor and detector nations and extracting seasonal patterns. Likewise, efforts have been made to harmonize the decision process whether to launch a RASFF notification with the EFSA RACE-tool, albeit only for chemical contaminants [6].

The use of RASNEX is exemplified with the fipronil contamination incident of 2017/2018. Fipronil contamination of eggs was caused by the illegal use of a detergent containing fipronil, which is prohibited in the EU for all food-producing animals, on poultry farms (laying hens) as a treatment against red lice (*Dermanyssus gallinae*). Since fipronil is transferred into chicken eggs [12–14], it made its way into egg products. Because of the vast quantity of produced eggs and globalized flows of goods and trade, those contaminated eggs and egg products spread rapidly across Europe and beyond. As a second example for the use of RASNEX, we processed RASFF notifications on cases of pathogenic bacteria (*Salmonella spp*.) and other microbial food safety hazards, e.g. *Enterobacteriaceae*, residues of veterinary medical products, as well as persistent organic pollutants like polychlorinated dibenzo(*p*)dioxins and furans (further referred to as “dioxins”).

## Materials and methods

**Fig 1:**
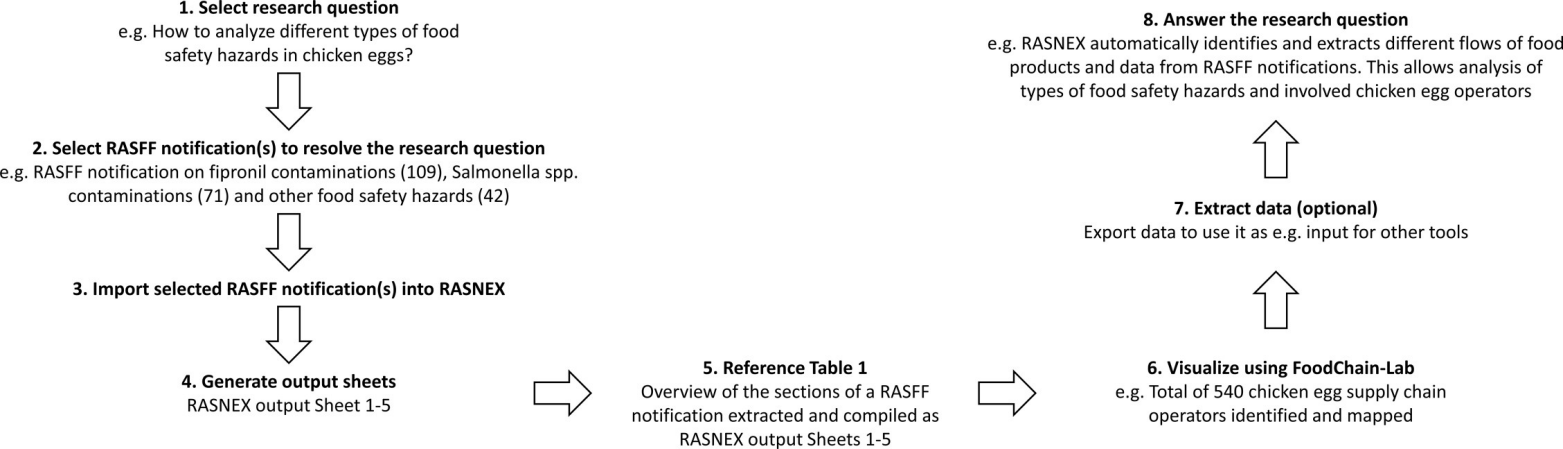
Methodological graphical scheme illustrating the functioning of RASNEX and the structure of this paper.

The methodological framework of RASNEX consists of several steps starting with choosing appropriate RASFF notifications to answering appropriate research questions (Fig 1).

### Programming environment

The Konstanz Information Miner (KNIME) [7, 15] framework was chosen as a programming environment to implement RASNEX, since it is centered on data mining and data science. KNIME allows users to graphically program by building so-called workflows that combine software modules with diverse purposes, called nodes, to process data [16]. Edges define the information flow between nodes. The result of the calculation of each node can be inspected at the node’s outport. Many KNIME nodes can be used within the FoodChain-Lab [17] environment centered around food and feed tracing. It is possible to import and combine many types of data into KNIME and to automatically create reports. In terms of risk management, the modular workflow-based approach [16] leads to a transparent, reproducible and transferable result. The KNIME platform includes different data analysis features allowing for applications in different research fields, e.g. microbial risk assessment and general data mining. The results can be integrated into workflows built by the community and further processing of the generated outputs is possible.

All KNIME nodes that have been used, including their source and function, are listed in NodePit, a search engine allowing for searching, finding and installing KNIME nodes (https://nodepit.com/).

### RASFF notification structure

RASFF notifications report new information on a health or nutritional risk case detected in one or more consignments of food or feed and are divided into three major sections dealing with the main products, the related products and additional information (follow-up information). RASFF notifications can be accessed via the RASFF portal. An account for the RASFF portal is required to download the PDF file of a notification. Those PDFs are not publically available because they contain confidential data on trade relations. Therefore, access is restricted to authorized users (e.g. risk assessors, risk managers and authorities). In the current version, RASNEX 1.0 cannot directly access the RASFF portal. Therefore, PDF files need to be manually downloaded by the user. The sections dealing with the main and related products include information such as the product name, the product category, the lot number and the net weight of the lot. RASFF notifications in addition contain relevant information about market actors such as producers, packers, retailers and traders. Lastly, RASFF notifications contain the physical addresses of market actors, enabling an overview and geographical mapping of those actors. Starting in 2011, the RASFF format was changed with the introduction of the iRASFF system, an IT platform used to share RASFF notifications among members [18]. RASNEX makes use of the new RASFF format.

### Using RASFF notifications as RASNEX case studies

RASFF notifications constitute the source of the workflow, providing information about contamination events. The tool RASNEX that we present here can be employed for specific cases, but it can equally be used for combined RASFF notifications related to a certain good (e.g. chicken eggs, pork, pet food, etc.). The RASFF notification used as one case study for RASNEX contains information on the fipronil contamination incident (RASFF notification 2017.1065, Notification number: 333999). As a second case study, we used RASNEX to analyze RASFF notifications on different food safety hazards identified in the flow of chicken egg products during recent years and listed in the analysis section of the RASFF notifications: fipronil (109 notifications), *Salmonella spp*. (71 notifications) and other types of food safety hazards (*Enterobacteriaceae*, residues of veterinary medical products and dioxins, combined as “Other”; 42 notifications).

### Selection of RASFF notifications to resolve a specific question

When using RASNEX with a combination of notifications (caused by several chemical, biological or other food safety hazards) to reach significant coverage of the flow of food or feed products, they need to be carefully selected in order to extract meaningful information regarding a concrete question or research problem. The preselection of RASFF notifications and their follow-ups should be performed, such that they contain data on market actors relevant to that very question. If the extraction of information on the chicken egg supply chain is of interest, it may be started by filtering within the RASFF by product name with the keyword “egg” and synonyms and proceeded by downloading all filtered notifications. This process excludes most true negatives. Later, the remaining notifications need to be classified into:

True positives: RASFF notifications solely regarding egg and egg-products that include only market actors of the flow of chicken eggs.

False positives: 1) Products like “eggplant” or “duck eggs” that contain the text “egg” but are not relevant or of less importance because they are not “chicken eggs”. 2) RASFF notifications regarding products that should not contain “egg”, for instance when an egg was used as an undeclared ingredient. 3) Notifications exclusively containing information on irrelevant market actors for our question, e.g. suppliers of synthetic food additives (such as E102 yellow in pasta and carotenoids/xanthophylls like canthaxanthin in compound feed for laying hens).

Mixed: RASFF notifications for chicken egg-containing products, which nevertheless contain information on market actors of related but not directly relevant supply chains. Examples: Sausages containing egg; eggs and meatballs supplied by the same actor.

True negatives were discarded in the first keyword-filtering step within the RASFF. False positives were likewise discarded. False positives needed to be identified and discarded manually by reviewing the filtered RASFF notifications and by deleting them from the folder containing all PDF files that were used as an input for RASNEX. All true positives were then analyzed using RASNEX. Mixed notifications were decided upon on an ad-hoc basis, making sure they contribute market information relevant to our specific question. Sorting out true negatives PDF files is currently manual but has the potential to be further automated, for example by building a separate KNIME workflow. This separate KNIME workflow might use whitelists with keywords, for example synonyms for the product of interest (in our case “egg”, “shell egg”, “chicken egg”, etc.). These whitelists need to be complemented by blacklists to exclude RASFF notifications with keywords that are not of interest (e.g. “eggplant”, “fish egg” or “duck egg”). There may be some false negatives excluded in each filtering step, leading to supply network information loss.

### Visualization using FoodChain-Lab

The KNIME extension FoodChain-Lab [17] is used to graph the actors of the supply network. FoodChain-Lab is a KNIME extension implemented and supported by the German Federal Institute for Risk Assessment (BfR). It can be downloaded and used as open software tool. Here, it is employed to match the actors stated in the RASFF notification with their Graphical Information System (GIS) coordinates for all extracted operators. Geocoding data was automatically retrieved from the German Federal Office for Cartography and Geodesy (BKG, www.bkg.bund.de), Photon (a geocoding tool hosted by BfR) and Bing (Microsoft, www.bing.com). Furthermore, FoodChain-Lab is used to add arrows to the links in the network map, visualizing the delivery routes of contaminated goods, identifying supplier and receiver in each linkage in the flow of products. Cartographic material from USGS National Map Viewer (https://viewer.nationalmap.gov/advanced-viewer/) was used for illustrating maps. Map services and data available from U.S. Geological Survey, National Geospatial Program.

### RASNEX input and output

RASNEX in its first released version (1.0) is available for download from https://doi.org/10.5281/zenodo.4322555 including the source code; documentation for running and installing the software; and a sample RASFF notification (dummy) PDF as test dataset. Competent authorities can download actual RASFF notifications and import them into RASNEX. RASFF notifications can be downloaded in PDF format using iRASFF. Ideally, the most recent RASFF notification should be used because it also contains all previously released information.

**Fig 2:**
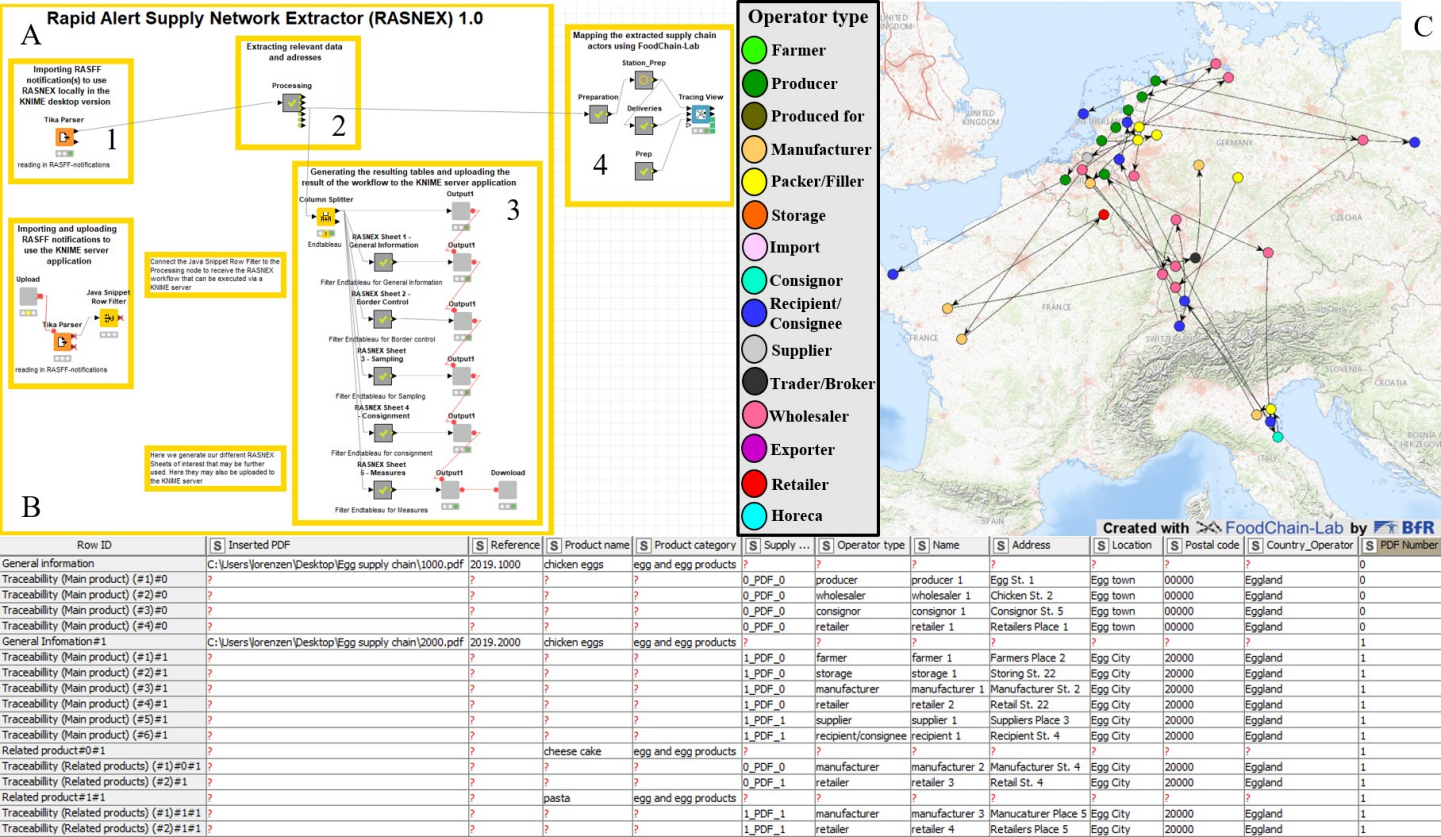
Function of RASNEX. (A) The RASNEX 1.0 workflow is divided into 4 parts: 1) Importing RASFF notification(s), 2) extraction, 3) mapping of operators involved in the different supply chains, 4) displaying the results on a webpage and generating the output. (B) Exemplary anonymized RASNEX 1.0 output sheet on the consignment providing information on the structure of different supply chains. (C) Graphical mapping of a chicken egg fipronil contamination incident from 2017/2018 using GIS showing 39 operators, which are part of ten different supply chains, located in seven central European countries and categorized into nine operator types.

RASNEX’ 1.0 output is divided into different sections performing different functions (Fig 2A). After converting the PDF format into the internal KNIME format, RASNEX 1.0 extracts related data in five output sheets containing information on 1) general information on the analyzed RASFF notification (e.g. notification number, date of notification), 2) border control 3) sampling (e.g. laboratory addresses), 4) consignment (e.g. operator types and addresses) and 5) taken measures (e.g. measures taken in and by). These output sheets are named according to the corresponding sections in the RASFF notifications form. The Column Splitter node named “Endtableau” aggregates all the information contained in the analyzed RASFF notification and enables users to build further applications based on their needs. Files that are not in PDF format, PDF files that are not RASFF notifications and RASFF notifications that use the old RASFF format are ignored by RASNEX 1.0, but will not stop it from extracting other valid files. Furthermore, a graphical mapping of all extracted operators contained in the imported RASFF notification is plotted onto a map (Fig 2C).

RASNEX 1.0 mines information from the two major chapters within a RASFF notification, the main and related products. For both, RASNEX 1.0 extracts the address of the involved supply chain actors (operators) in chronological order from the beginning of the food or feed supply chain to its end, as well as the country to which those products were distributed (Fig 2B). The workflow offers print numbers (in the case of shell eggs) or lot numbers related to the treated main or related product. Furthermore, information such as the total net weight of the lot or the result of laboratory analyses is extracted from the RASFF notification and made available for further data analysis. The product name, product category and the type of contamination are extracted. This information is utilized to understand the distribution of a contaminated product through each food or feed supply chain in case of a contamination event. The resulting sheets can be exported e.g. as Excel files. As shown in Table 1, distinctive parts of the RASFF notification are extracted.

**Table 1:** Overview of the sections of a RASFF notification extracted and compiled as RASNEX 1.0 output Sheets 1–5.

Section	Section classification	Extraction Status	Example of extracted information	RASNEX Output Sheets
1. General Information	Standardized	Extracted	Notifying Country	RASNEX Sheet1 –General Information
2. Distribution Status	Standardized	Extracted	Distribution to other member countries	RASNEX Sheet 1 –General Information
3. Countries notified for the notification and associated follow-up	Standardized	Extracted	Germany	RASNEX Sheet 1 –General Information
4. Countries notified for the notification and associated follow-up	Standardized	Extracted	Germany	RASNEX Sheet 1 –General Information
5. Risk	Standardized	Extracted	Serious risk	RASNEX Sheet 1 –General Information
6. AAC (Administrative Assistance and Cooperation)	Standardized	Extracted	Serious	RASNEX Sheet 1 –General Information
7. Products	Standardized	Extracted	Product name, Sampling, Operator, Measures	RASNEX Sheet 2 –Border Control
				RASNEX Sheet 3 –Sampling
				RASNEX Sheet 4 –Consignment
				RASNEX Sheet 5—Measures
8. Related products	Standardized	Extracted	Product name, Sampling, Operator, Measures	RASNEX Sheet 2 –Border Control
				RASNEX Sheet 3 –Sampling
				RASNEX Sheet 4 –Consignment
				RASNEX Sheet 5—Measures
9. Additional information	Variable/not standardized	Not Extracted	Variable and unformatted	No output (not extracted)
10. Attached documents	Variable/not standardized	Not Extracted	Variable and unformatted	No output (not extracted)

The eight standardized sections of the RASFF notifications (1–8) contain various types of information about the ongoing contamination event. Section 7, for example, contains information about the product (main product) covered by the RASFF notification, containing product name and category, analytical results and traceability information and taken measures. Traceability information is, in this case, the distribution status, a lot number, background information and involved operators (Table 1). All the information contained in sections 1–8 is completely extracted by RASNEX 1.0. Section 9 and 10 contain additional information and attached documents whose format is not standardized and is therefore not currently analyzable.

The second function of RASNEX is the graphical mapping of all known actors of the food or feed supply network contained in the RASFF notification onto a world map (Fig 2C). The mapping of the operators of the supply chain requires assigning geographical coordinates to their extracted addresses. The Tracing View node is used to graphically map all operators of the supply chains onto a world map. The different operators are illustrated as circles on the map. The color of the circle can be used to indicate the type of actor (e.g. producer, wholesaler, manufacturer or retailer) or the type of hazard (e.g. fipronil, *Salmonella spp*.). Arrows connect the different stations of the supply chain, pointing in the direction of the flow of goods. Each of the supply chains is illustrated by a starting point (circle with only one starting arrow), several actors of the supply chain (connected through arrows pointing to the following station) and an end point (circle with only an incoming arrow). The automated mapping allows risk managers to obtain a continuously updateable overview of a currently ongoing contamination event.

## Results

### Analyzing the fipronil contamination incident of 2017/2018 using RASFF notification 2017.1065

In the first use case, we analyze the European fipronil contamination incident from 2017/2018, in which a total of 721 RASFF notification follow-ups referring to the original notification on fipronil (RASFF notification 2017.1065) were generated. We analyzed the most recent RASFF notification using RASNEX 1.0 to make use of all available information on this contamination event. Here, we showcase the automated data extraction from RASFF notifications using RASNEX. The resulting RASNEX output sheet 3 (Consignment) has 23 rows for the main product “eggs”. For this main product, the workflow identifies five different flows of food products contained in the RASFF notification. Flows of food products, as a part of related supply chains, in this case means that the eggs were delivered through four different paths (since for the fifth flow of products only the consignment number is available). The chains start at four different producers. The eggs are delivered to different receivers, e.g. packers/fillers, wholesalers, suppliers, retailers, manufacturers or traders/brokers. The length of the flow of products differs in the example of the fipronil contamination incident and ranges between two and five chain links. Furthermore, five flows of products on related products (e.g. liquid egg and egg yolk powder) and related information (e.g. analytical results, involved operators and taken measures) were extracted. The information contained in the output sheet on the consignment was used to plot a graphical mapping of all extracted operators onto a world map (Fig 2C).

### Analyzing different types of food safety hazards in chicken eggs

In a second use case, a broader set of food safety hazards in the egg supply chain was used to illustrate a larger fraction of operators reported as involved in food safety hazards and to illustrate inconsistent reporting behaviors between member countries. Besides the fipronil contamination incident of 2017/2018, we analyzed a set of contamination of eggs with fipronil, *Salmonella spp*. as well as “others” (e.g. dioxins, *Enterobacteriaceae* and residues of veterinary medical products). Analyzing the egg supply network by its hazard types granted further insights. In the first run, we analyzed all known actors of the supply network that were involved in fipronil contamination incident in eggs (109 analyzable RASFF notifications) during recent years. Second, all available *Salmonella spp*. contaminations in eggs (71 analyzable RASFF notifications) were analyzed. Third, all known other food safety hazards (42 analyzable RASFF notifications) in the egg supply network were analyzed.

**Fig 3:**
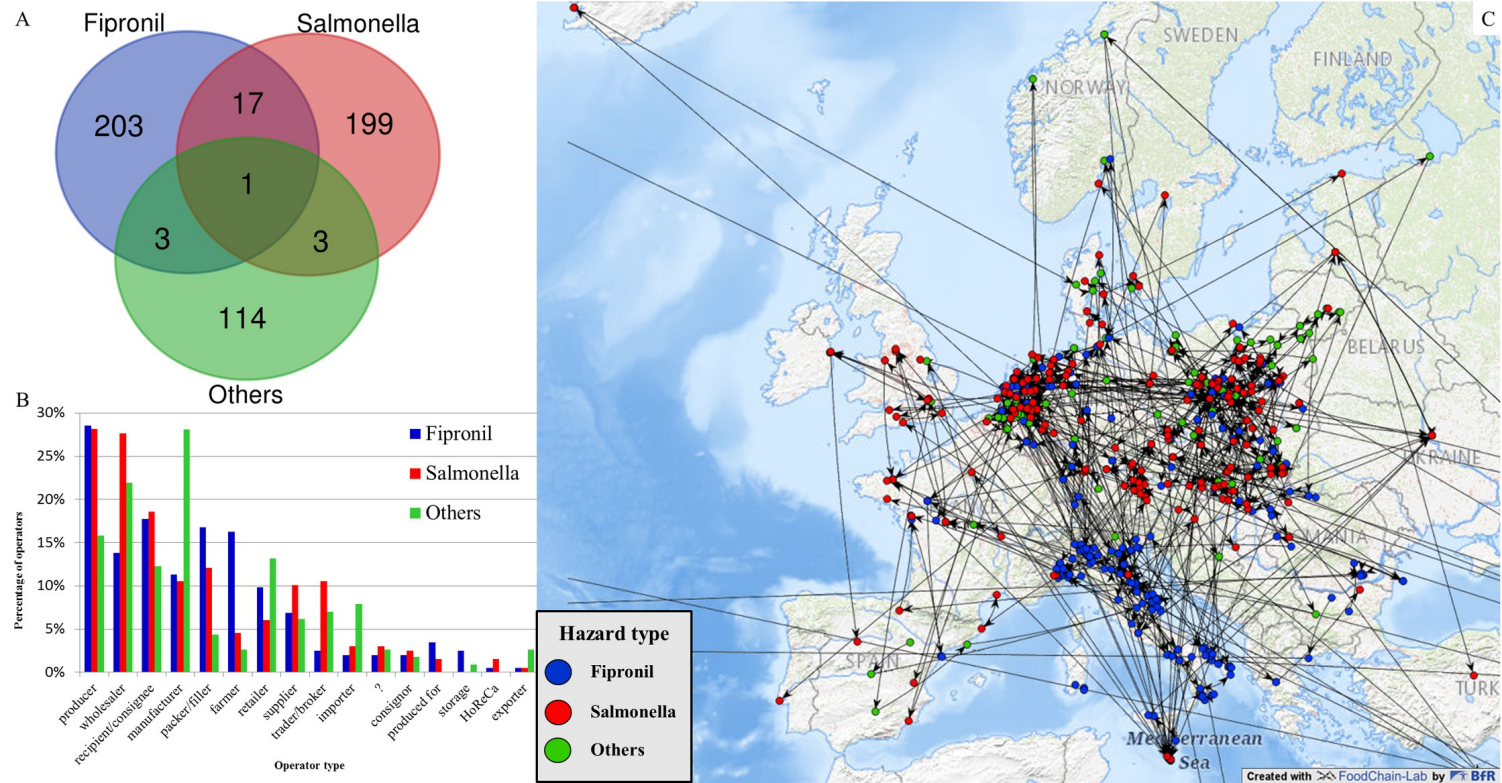
**Three types of food safety hazards in the chicken egg supply network analyzed by RASNEX 1.0 from 222 RASFF notifications: fipronil (blue), *Salmonella spp*. (red) and other contamination events (green).** (A) Venn diagram indicating the number of operators involved in one hazard type as well as their intersections (numbers involved in more than one of those food safety hazards). (B) Distribution by operator type involved in each hazard in the chicken egg supply network. Columns by contamination type add up to more than 100% since some actors appear as different operator types. (C) Geographic Information System (GIS) map for each hazard type in the European chicken egg supply network. It comprises 540 unique operators, which are part of different supply chains and are located in seven Central European countries.

All three types of contamination events were compared with each other. A Venn diagram [19] is generated, revealing the amounts of involved operators in the three hazard types and their overlays (Fig 3A).

The largest number of unique involved operators (203) is found in the fipronil contamination incident, followed by *Salmonella spp*. (199 operators) and other food safety hazards (114 operators) (Fig 3A). Total numbers of all operators in all supply chains (including those that occur in more than one chain) are 277 for fipronil, 279 for *Salmonella spp*. and 145 operators in RASFF notifications on other food safety hazards. Only 24 out of 540 unique operators referenced in the RASFF notifications are involved in more than one type of contamination event (Fig 3A). Most of the intersection occurs between operators involved in fipronil as well as *Salmonella spp*. contamination events. The operators occurring in more than one hazard type can be classified as large businesses with international impact. Six of them are classified as recipients/consignees, six are wholesalers, four are manufacturers, three are producers, two are suppliers, one each is a retailer, importer and classified as “produced for”.

### Analyzing different types of involved operators

Analyzing relationships between operators (basic scrutiny of network parameters made using the Network Analyzer node of KNIME) showed that about 30 per cent of all operators extracted from the RASFF notifications were a starting point of related flows of products. In contrast, operators involved in more than one hazard type were primarily located in the middle or at the end of their supply chain.

The plot of types of operators (Fig 3B) involved in each hazard reveals a trend for notifications being more often triggered at the level of producers, recipients/consignees, packers/fillers and farmers. Put differently, RASFF notifications tend to be generated in the upstream section of the egg supply chains. Analyzing the involved 15 different types of operators of the egg supply network shows that especially producers, wholesalers, recipients/consignees, manufacturers and packers/fillers are affected by contamination events (Fig 3B). A question mark characterizes 13 operators for which there is no information about their type in the notifications (Fig 3B). This reveals a limitation of RASFF, namely that it is possible to leave important fields blank. RASNEX 1.0 is only able to mine information that is shared by RASFF members and added to the corresponding fields. A considerable number of farmers are documented in RASFF as being involved in fipronil contamination events, which exceeds by more than a factor of three those documented as involved in *Salmonella spp*. contamination events. There is a concentration of farmers reported as being involved in fipronil contamination events in Italy while only few are reported as affected by *Salmonella spp*. (Fig 3C). *Salmonella spp*. cases were especially reported at the wholesalers and producers level (Fig 3B). Other food safety hazards are most often reported for wholesalers and manufacturers. Only few importers are reported to be involved in fipronil and *Salmonella spp*. contamination events. Interestingly there are relatively few RASFF notifications reporting a hazard with retailers being involved. If retailers are involved, they are mostly mentioned in attached documents.

### Analyzing parts of the European chicken egg supply network

**Fig 4:**
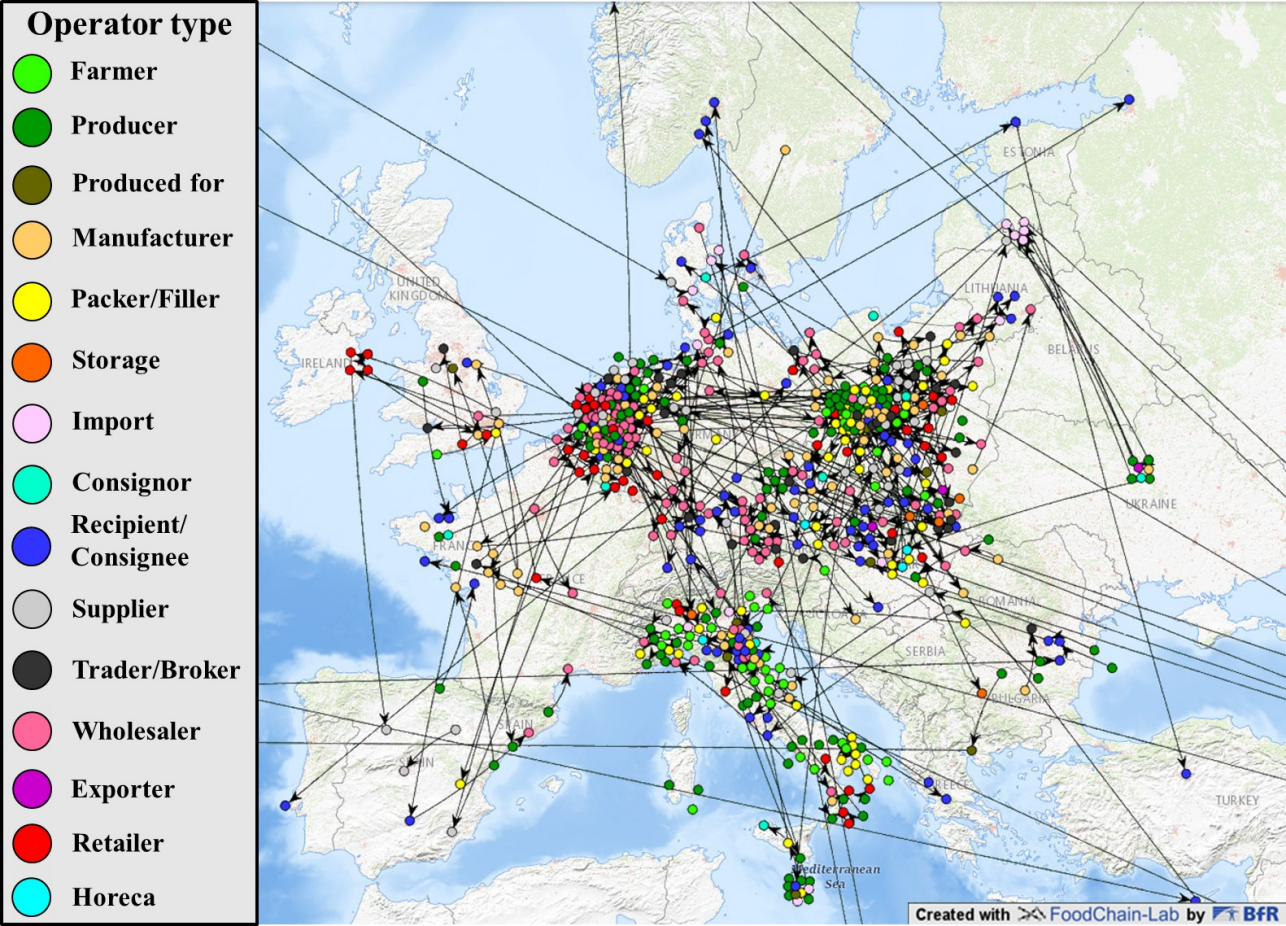
Categorization into 15 operator types (color-coded) using RASNEX 1.0 to analyze 222 RASFF notifications mapped using a Geographic Information System (GIS). It visualizes a total of 540 unique operators of various flows of food products, located in 40 countries and comprises information contained in RASFF for several food safety hazards on operators reported to be part of a European chicken egg supply network.

In a last step, we use RASNEX 1.0 to gain more information on the different operator types that are involved in several food safety hazards in the egg supply network. RASNEX 1.0 can analyze and map supply networks of distinct products in a Geographic Information System (GIS) using OpenStreetMap. All RASFF notifications available for the product chicken egg were used as input for RASNEX 1.0. The RASFF portal was filtered for the subject “egg” to extract all available information on the European egg supply network. This resulted in 706 RASFF notifications on various food safety hazards and contamination events in eggs. As described in the Section “Selection of RASFF notifications to answer a specific question”, we need to filter to find true positives. This led to 403 RASFF notifications on chicken egg supply chains. Among those, 222 notifications are in the current RASFF format and are therefore analyzable with RASNEX 1.0. Out of these 222 RASFF notifications, RASNEX 1.0 extracts within a few CPU-minutes more than 176,000 pieces of detailed information and compiles the 5 output sheets. Plotting the corresponding map (Fig 4) extends the already known network of the fipronil contamination incident of 2017/2018 (Fig 2C).

A total of 540 unique operators organized in 278 supply chains are extracted from the 222 notifications on different food safety hazards in chicken eggs. The map shows an accumulation of wholesalers and retailers in the Netherlands that were involved in contamination events in the chicken egg supply network. There are also many affected producers in Poland, which is consistent with the known fact that Poland is one of the major European egg producers [20]. In comparison, France, Spain and UK, which are also major European egg producers, are clearly underrepresented [20].

## Discussion

### Analyzing different types of involved operators

The sheer size of big players may suffice to explain their involvement in multiple contamination events (e.g. involvement in *Salmonella spp*. as well as fipronil contamination cases, Fig 3). Despite the limited number of analyzed operators, it seems that the position in the supply chain has an influence on the involvement in multiple contamination events. Most operators occurring in more than one type of hazard are pooling eggs, which means that they receive large quantities from other operators and either fulfil the function of a commercial packing center or they process eggs. Therefore, operators closer to the consumer seem more likely to be involved in multiple contamination events. Another plausible explanation would be a higher probability for big players to occur in RASFF notifications due to more resources dedicated for quality control. If this is the case, it follows that there will be a serious underreporting of smaller operators. Here, further investigation on the attached documents would be helpful to analyze all operators reported in the RASFF notifications. This is planned for future versions of RASNEX.

### Analyzing the reporting behaviors of different member countries

RASNEX allows displaying of several types of contamination on a map. Mapping fipronil, *Salmonella spp*. and other contamination cases shows clear regional differences in the spreading of the different types of contamination and/or of its reporting (Fig 4). The map shows a denser clustering of operators reported as involved in fipronil contamination events in Italy. This is likely due to inconsistent reporting behaviors among member countries. RASFF notification 2017.1065 mostly contains information related to German egg supply chains. There were only few notifications dealing with fipronil in German egg supply chains besides this one (two other notifications released by Germany). In contrast, there were 63 notifications released by Italy dealing with fipronil in eggs. Due to the limited number of supply chains added to the relating consignment section of the RASFF notification, RASNEX 1.0 extracts more operators the more notifications are released. This impression of Italy reporting highly on fipronil in eggs confirms the information found in the country fact sheets for Italy, Germany and the Netherlands published by the European Commission for 2017 and 2018 (https://ec.europa.eu/food/safety/rasff/country-fact-sheets_en). As shown in the facts sheets, only in Italy is fipronil among the “top 5 hazards” (71 notifications in 2017). Nevertheless, the impression that there are more fipronil cases in Italy than in other European countries may not hold true. This effect is mainly caused by inconsistent reporting behaviors. Whilst Italy released several individual notifications on mostly regional flows of products, other countries (especially Germany, Belgium and the Netherlands) shared most information related to fipronil using a single RASFF notification (2017.1065). The latter contains more than 260 pages and more than 900 attached documents. Manual analysis of all 109 RASFF notifications on fipronil contamination events revealed that 71 out of 109 notifications did contain operator and supply network information; interestingly, all such notifications were from Italy. Taking the attached information of RASFF notification 2017.1065 into account, a much denser network especially in Germany, Belgium, Denmark, Czech Republic and the Netherlands would be discovered.

A second striking aspect is the modest number of Italian operators reported as involved in *Salmonella spp*. contamination events. The country fact sheets provided by the European Commission for Italy also reveal there are less *Salmonella spp*. contamination events reported by Italy compared to Germany (about one third) and the Netherlands (about one fourth) in 2017 (for all reported products, not only eggs).

We posit two plausible explanations for the concentration of mapped operators involved in fipronil contamination events around Italy: Either fipronil contamination events occur more frequently in Italy than in other European countries, or Italian competent authorities more frequently report (fipronil) contamination events in comparison to other member countries. At face value, the RASNEX analysis of a part of the European chicken egg market seems to reveal an acute problem in Italy and a high frequency of fipronil contamination in eggs. Instead, this is likely related to a selection bias caused by the used information from RASFF, which is profoundly influenced by the inconsistent reporting behaviors of the member countries. It has in fact been shown that Italy uses RASFF intensively, generating 377 original notifications and more than 750 follow-up notifications in 2019 [4]. Comparing our findings to official perceptions of fipronil contamination events enhances the value of the perspective RASNEX delivers: Whilst official and public perceptions focused on fipronil contamination in the Netherlands, Belgium and Germany [21], RASNEX reveals a concentration of reported operators involved in fipronil contamination events in Italy. Further analysis has shown Italian RASFF notifications were mostly related to other Italian operators, so that the concerned products never left the country. This points to more nationally oriented flows of eggs in Italy compared to e.g. the Netherlands. Self-sufficiency rates (100% in Italy vs. 308% in the Netherlands, 2013), further reinforce this impression [22].

Our results suggest that some countries tend to report less in RASFF than others. In Spain, there are only few operators mapped, whereas in Italy there is a large number of mapped actors in our analysis (Fig 4). Southwestern European countries (Portugal (42), Spain (279) and France (248)) tend to generate fewer original notifications compared to Germany (534), Italy (377) or the Netherlands (378) (using the number of original notifications by member countries in 2019 [4]). This may be caused by less frequent contamination, less stringent measures, cultural tendencies to underreport or most likely inconsistent reporting behaviors due to different incentives and motivations to launch a RASFF notification.

### Advantages and challenges using RASNEX

The RASFF system was set up to support the free movement of safe and wholesome foods within the European single market (Reg (EC) 178/2002) and as a health risk data collection and analysis system. In its current inception, however, the use of RASFF data is mostly limited to its immediate purpose as an alert. RASNEX allows for the extraction of data stored in the system and enables additional analysis. For ongoing contamination events (using one RASFF notification) RASNEX extracts the data sheets and the associated geographical map within seconds. Feeding in large amounts of data from several chemical contaminants or biological agent outbreaks provides comprehensive insight into reported alerts and existing reporting behaviors of different member countries and reported flows of goods. For this use case, hundreds of RASFF notifications may be used. Processing time depends on the exact number and contained information. Processing 222 RASFF notifications took about 30 CPU-minutes using two cores of a 2015-released Intel Core i3-6100. RASNEX is implemented in the open-source analytics platform KNIME and also works on a KNIME server and therefore can be extended with and used as an input by other tools. The output from RASNEX can be processed using the BfR FoodChain-Lab environment as well as be used as an input for other risk management applications. One example is the usage of the EFSA RACE tool [6] for retrospective risk evaluation using past RASFF notification analytical results (in an extension of the RACE use case intended by EFSA, prospective decision-making to report in RASFF). Laboratory analytical results can be extracted from RASFF using RASNEX and then be used as an input for the EFSA RACE tool. In this way, past RASFF notification analytical results can be compared to current legal limits and expected exposure. Following revealed and visualized flows of products from farm-to-fork, further tools may be implemented to infer contaminant flows between different actors of the supply chain. One example is toxicokinetic feed-to-food transfer models, for example for fipronil transferred into eggs [14]. Such linkage of analysis tools allows risk managers and risk assessors to use processing factors for example for fipronil in egg yolk to better define areas of risk and to focus management activities on critical parts of the supply chain. It is possible to develop and implement further tools for other use cases for the customer (e.g. Tools for Quantitative Microbial Risk Assessment to aid in food-borne epidemiological investigations). By uncovering patterns of operator behavior and origin of hazards, users of RASNEX can meet the demands on risk prediction framed by Reg (EC) 178/2002. It may be used to visualize flows of goods within and between countries. Country-specific tools set up on the basis of data shared through RASFF and extracted by RASNEX can decrease reaction times in case of a contamination and help to safeguard supply chains. RASNEX is updatable, automates the extraction of information contained in RASFF notifications at hand, is much faster than manual extraction of the related data, is integrative and may be used as an input for further data processing and gathering.

A limitation of RASNEX 1.0 is its input, the RASFF notification. The workflow is built to extract food and feed supply chain data from the standardized fields in RASFF notifications. It is only possible to extract data from the positions (fields) intended to contain food and feed supply chain data. Because the structure of the RASFF notification is standardized, those sections containing relevant information have consistent formatting. Those are mainly the “Products” and the “Related products” sections. Information added in follow-up notifications (fups) to these sections can be extracted, whilst information added to the chapter “Additional information”, mostly as free text, cannot be extracted at this stage of development. This is because this section has arbitrary content and format, often containing references to information contained in attached documents. Future usability of this section may require machine learning and text mining to further improve RASNEX. Of higher priority instead is the extraction of information directly contained in the attached documents. The non-standardized attached documents are a limitation for RASNEX 1.0 because they cannot yet be analyzed and extracted automatically. Therefore, the next planned improvement of RASNEX 1.0 is the extraction of information contained in selected types of attachments with a more or less standardized structure (e.g. delivery documents, bills).

Using RASNEX 1.0, only information shared through the RASFFF system can be extracted at this stage of development. Extraction of information and the visualization of ongoing and past contamination events is the intended functionality of RASNEX. When combining information from several notifications (as in the second use case), a bias in the supply network is evident. In our study, we could show how this bias reveals information about reporting tendencies for the egg supply chain, especially for the southwest of Europe (Fig 4).

### Limitations of the RASFF system

Our findings clearly show that there are inconsistent reporting behaviors between member countries. This leads to more mapped actors of flows of egg in e.g. Italy, Poland and the Netherlands and a clear underrepresentation of flows of eggs in southwestern Europe (Spain, France). RASFF Standard Operating Procedure (SOP) makes the following provision: “The assessment whether or not there is a risk involved in non-compliant food/feed, and whether the risk is such as to require the notification to the RASFF is the responsibility of the members of the network” [3]. It may be beneficial to standardize the decision-making process on the generation of RASFF notifications as implemented in the EFSA RACE-tool in order to make sure that it is uniform and transparent.

A key limitation of RASFF is its different handling by its members, leading to inconsistent reporting behaviors, which may be partly due to their different political systems (federal vs. unitary states). The dependence of RASFF on human decisions leads to variable amounts of contained information in the related fields of the notification and attached documents. Computer-guided procedures and decisions, as pioneered by the EFSA RACE-tool to harmonize the decision to launch a RASFF notification may also help harmonize the information contained within RASFF notifications in the future. This can be enabled by on-site training courses, such as those offered for the EFSA RACE-tool.

An explicit and unique food classification system like EFSA FoodEx2 [23] may help better filter for RASFF notifications on particular products, e.g. declaring chicken egg consistently in all RASFF notifications provided as “chicken egg” with a unique classification instead of only “egg”. Consistent wording should also be applied in the attached documents and the additional information section of the RASFF notifications to improve future ability to extract information from these sections. This would reduce the amount of false positive filtering because products containing the word “egg”, e.g. “eggplant” or “duck egg”, would not be filtered in.

Another limitation when visualizing flows of products using RASNEX 1.0 is the limited chain length due to the RASFF SOP WI 3.1 [24] allowing for only up to five operators with full details in each chain. Due to its intended usage, only operators involved in contamination events are included in the RASFF notifications, so that RASNEX 1.0 may not show all operators active on the market. Visualization is limited to reported flows of products in case of ongoing contamination events and to reported actors of supply networks when using multiple RASFF notifications, leading to a visualization ultimately subject to the reporting behaviors of member countries.

## Conclusion and implications

We have built a workflow in KNIME that is able to extract food and feed supply chain data from previously downloaded RASFF notifications and to summarize this data in sheets and using a geographic visualization. RASNEX enables the user to obtain and maintain an overview of ongoing contamination events and to visualize parts of supply networks reported to have been involved in contamination events. RASNEX can be used as a decision-support tool for risk managers during ongoing contamination event. RASNEX also provides sheets with all the information contained in the analyzed RASFF notifications (currently except for the “Additional information” section). In this way, RASNEX can be used as an input for further tools to analyze the data contained in the RASFF notifications (for example laboratory analytical results).

We used RASNEX 1.0 to reconstruct the flows of eggs contained in the corresponding fields of the RASFF notification on the fipronil contamination incident of 2017/2018 (RASFF notification 2017.1065, Notification number: 333999) to showcase the usability of RASNEX to support the resolution of ongoing contamination events. Based on 222 RASFF notifications on eggs and egg products, we have visualized an egg supply network. In this way, we extended the egg supply network extracted from the fipronil contamination incident of 2017/2018. The reproduced supply network nevertheless does not represent the complete egg supply network because the input data is based only on operators reported to be involved in contamination events via the RASFF system. Therefore, we visualized only parts of the egg supply chain and the inconsistent reporting behavior of member countries. This allows for a critical questioning of the RASFF system pointing out potentials for improvement. We have shown that there are regional differences in the amounts of operators reported to be involved in fipronil, *Salmonella spp*. and other contamination events with clusters in Italy, Poland and Benelux. This may be due to the inconsistent handling of the RASFF-system by its members and the different amounts of notifications and contained information in the related fields of the notifications. The EFSA RACE-tool may help as decision-making tools in the future to harmonize decisions to launch a RASFF notification, which would in turn be reflected in a more uniform picture across flows of European food and feed products with RASNEX. We have shown that few operators were reported to be involved in more than one type of contamination. The size of those players and their central position in the supply chain may suffice as an explanation for their involvement in multiple contamination events. An alternative explanation is the inconsistent reporting behavior among member countries. We have shown the need for explicit and unique product identifiers for better filtering of relevant RASFF notifications and in order to enable future data extraction from the additional information section and the attached documents.

For future research, we suggest analyzing other relevant food or feed supply chains. Extracted supply chain information may be further used to synchronize with other available data sources to enhance mapped supply networks. Network analysis may be applied to further deepen insights into existing supply network structures. Deeper extraction of the information in the attached documents to obtain more detailed insights is planned for future versions of RASNEX.

RASNEX provides risk managers and assessors with instant windows on ongoing contamination events and provides a long-term overview of reported flows of food and feed products. It does so through raw data as well as graphical updateable maps based on the extracted information from the related RASFF notification(s). RASNEX helps users ensure more rapid resolution of chemical contamination and biological outbreak events to minimize impact on human health, animal health and finances. RASNEX 1.0 is available for download from Zenodo at https://doi.org/10.5281/zenodo.4322555 including source code, documentation and a test dataset.

## References

[1] Commission E. Regulation (EC) No 178/2002 of the European Parliament and of the Council of 28 January 2002 laying down the general principles and requirements of food law, establishing the European Food Safety Authority and laying down procedures in matters of food safety. Official Journal of the European Communities. 2002;31(01/02/2002):1–24.

[2] European Commission. Regulation (EC) No 183/2005 of the European Parliament and of the Council of 12 January 2005 laying down requirements for feed hygiene. Official Journal of the European Union. 2005:1–22.

[3] European Comission. Standard operating procedures of the Rapid Alert System for Food and Feed (RASFF) and the Administrative Assistance and Cooperation (AAC) networks. In: Directorate G: Veterinary and International affairs Unit DDG2. G4.: Food asat, editor.: European Commission Health and Consumers Directorate—General; 2018. p. 1–51.

[4] European Comission. RASFF, the Rapid Alert System for Food and Feed 2019 annual report. Publications Office of the European Union Luxembourg; 2019. p. 1–56.

[5] Weiser AA, Gross S, Schielke A, Wigger J-F, Ernert A, Adolphs J, et al. Trace-back and trace-forward tools developed ad hoc and used during the STEC O104:H4 outbreak 2011 in Germany and generic concepts for future outbreak situations. Foodborne Pathog Dis. 2013;10(3):263–9. Epub 2012/12/26. doi: 10.1089/fpd.2012.1296 . 23268760 PMC3698685

[6] Fürst P, Milana MR, Pfaff K, Tlustos C, Vleminckx C, Arcella D, et al. Risk evaluation of chemical contaminants in food in the context of RASFF notifications. EFSA Supporting Publications. 2019;16(5). doi: 10.2903/sp.efsa.2019.EN-1625

[7] Berthold MR, Cebron N, Dill F, Gabriel TR, K\ T, \#246, et al. KNIME—the Konstanz information miner: version 2.0 and beyond. SIGKDD Explor Newsl. 2009;11(1):26–31. doi: 10.1145/1656274.1656280

[8] Parisi S, Barone C, Sharma RK. Chemistry and Food Safety in the EU: The Rapid Alert System for Food and Feed (RASFF): Springer; 2016.

[9] De Leo F, Coluccia B, Miglietta PP, Serio F. Food contact materials recalls and international trade relations: an analysis of the nexus between RASFF notifications and product origin. Food Control. 2021;120:107518.

[10] Soon JM. Application of bayesian network modelling to predict food fraud products from China. Food Control. 2020:107232.

[11] Petroczi A, Nepusz T, Taylor G, Naughton D. Network analysis of the RASFF database: a mycotoxin perspective. World Mycotoxin Journal. 2011;4(3):329–38.

[12] France. Draft assessment report on the active substance fipronil prepared by the rapporteur Member State France in the framework of Council Directive 91/414/EEC, April 2004. 2004.

[13] France. Final addendum to the draft assessment report on the active substance fipronil prepared by the rapporteur Member State France in the framework of Council Directive 91/414/EEC, Part 2 compiled by EFSA, January 2006. 2006.

[14] Gerletti P, Von Kleist M, Mielke H, Kuhl T, Pieper R, Lahrssen-Wiederholt M, et al. Transfer kinetics of fipronil into chicken (Gallus gallus domesticus) eggs. Computational Toxicology. 2020:100131.

[15] Berthold MR, Cebron N, Dill F, Gabriel TR, Kötter T, Meinl T, et al., editors. KNIME: The Konstanz Information Miner. Data Analysis, Machine Learning and Applications; 2008 2008//; Berlin, Heidelberg: Springer Berlin Heidelberg. doi: 10.3171/2018.8.FOCUS18325

[16] Falenski A, Weiser AA, Thöns C, Appel B, et al. Towards a Food Safety Knowledge Base Applicable in Crisis Situations and Beyond. BioMed Research International. 2015;2015:11. doi: 10.1155/2015/830809 26247028 PMC4515494

[17] Weiser AA, Thöns C, Filter M, Falenski A, Appel B, Käsbohrer A. FoodChain-Lab: A Trace-Back and Trace-Forward Tool Developed and Applied during Food-Borne Disease Outbreak Investigations in Germany and Europe. PLOS ONE. 2016;11(3):e0151977. doi: 10.1371/journal.pone.0151977 26985673 PMC4795677

[18] European Commission. The Rapid Alert System for Food and Feed‐2011 Annual Report. 2011:1–52.

[19] VIB/UGent Bioinformatics & Evolutionary Genomics. Calculate and draw custom Venn diagrams [cited 2019 12.12.2019]. Available from: http://bioinformatics.psb.ugent.be/webtools/Venn/.

[20] Bundesanstalt für Landwirtschaft und Ernährung. Bericht zur Markt- und Versorgungslage Eier 2019. Bonn: Bundesanstalt für Landwirtschaft und Ernährung 2019. p. 97.

[21] Boffey D. Contaminated eggs: Netherlands failed to sound alarm, says Belgium The Guardian. 2017.

[22] Bundesanstalt für Landwirtschaft und Ernährung. Bericht zur Markt- und Versorgungslage Eier 2017. Bonn: Bundesanstalt für Landwirtschaft und Ernährung 2017. p. 82.

[23] Authority EFS, Ioannidou S. EFSA Catalogue Browser User Guide. EFSA Supporting Publications. 2019;16(11):1726E. doi: 10.2903/sp.efsa.2019.EN-1726

[24] European Comission. RASFF WI 3.1 2018. Available from: https://ec.europa.eu/food/sites/food/files/safety/docs/rasff_reg-guid_sops_2018_wi-3-1_en.pdf.

